# The Effects of Indoor Pollutants on Health Care Workers, Patients, and Caregivers in Dental Clinics: A Systematic Review

**DOI:** 10.1055/s-0045-1813749

**Published:** 2026-01-23

**Authors:** Giulia Tetè, Manlio Santilli, Natasha Cinta Vinskid, Fabia Profili, Giuseppe Tafuri, Gianmaria D'Addazio, Bruna Sinjari

**Affiliations:** 1 “Sustainable Blue Economy and One Health”—XL Cycle, Department of Human Sciences, Law, and Economics, “Leonardo da Vinci,” UNIDAV, Telematic University, Chieti, Torrevecchia Teatina, Italy; 2 Unit of Prosthodontics, Department of Innovative Technologies in Medicine and Dentistry, University “G. d'Annunzio” of Chieti-Pescara, Chieti, Italy; 3 Hi-Tech Dental Materials Laboratory, Department of Innovative Technologies in Medicine and Dentistry, University “G. d'Annunzio,” Chieti-Pescara, Chieti, Italy; 4 Unit of Biomedical Science, Department of Biomedical Engineering, Hasanuddin University, Makassar, Indonesia

**Keywords:** indoor air quality, pollutants, volatile organic compounds, PM _10_, PM _2,5_, PM _5_, CO _2_

## Abstract

Most of the pollution inside a dental clinic comes from the external environment; therefore, the location of the building affects the air quality, as well as the work activity and the type of natural or mechanical ventilation. In the dental sector, pathologies caused by pollutants are increasing, mainly because of methyl methacrylate, 2-hydroxyethyl methacrylate, ethylene glycol dimethacrylate, and triethylene glycol dimethacrylate. However, there are still gaps in the literature regarding the potential effects of all environmental pollutants, and particularly the long-term effects on healthcare workers. A comprehensive search was conducted across PubMed, Embase, Web of Science, and Cochrane Library databases, without time limits, resulting in a total of 155 scientific articles. After the removal of the duplicates, 86 single papers remained for further analysis. The titles of these articles were manually reviewed to include relevant references related to the presence of indoor pollutants in the air of dental clinics. Following this screening process, 10 studies were identified as relevant to the topic of the systematic review. Seven scientific articles were selected to be included in this review. The seven experimental studies reported various air pollutants related to diseases affecting dental health. In particular, the levels of volatile organic compounds, carbon dioxide, and temperature were analyzed in a university dental clinic. Levels of environmental pollutants are much higher during working hours, particularly during dental procedures such as prosthetic and conservative dentistry, due to the chemical nature of the materials used. However, no study reported exceeding the limits set by national environmental regulations. Due to the heterogeneity of the studies, the variety of molecules, the variety of clinical facilities and their geographical location subject to different regulations, as well as the variety of measurement methods, including the variety of traditional and/or technological ventilation systems used in dental departments, a meta-analysis was not performed. Despite the limitations of this systematic review, it was possible to identify some key points that are useful for further in vivo studies aimed at developing specific guidelines to protect health care workers.

## Introduction

The European Union's ambient air quality directives require the measurement of concentrations in ambient air of a few pollutants that are considered to have significant effects on human health and the environment. Legal standards (i.e., maximum levels that must not be exceeded) exist for the following pollutants: sulfur dioxide, nitrogen oxides and nitrogen dioxide, particulate matter (both PM _10_ and PM _2.5_ ), lead, benzene, carbon monoxide, ozone, arsenic, cadmium, nickel, and benzo(a)pyrene. 1 In fact, global pollution is responsible for at least one in five deaths worldwide. 2 Most of what we know about pollution relates to the external environment, but most of the population spends their lives in buildings, both for work and for leisure. 3 Despite growing interest from the research community due to the COVID-19 pandemic, indoor pollution is less regulated than outdoor pollution. This is due to the limited amount of scientific evidence on the subject. Conducting research on indoor pollution requires significant economic, technical, and human resources. 4 Despite these obstacles, recently researchers are committed to finding solutions to make the air inside buildings harmless to human health. 5 There is very little scientific evidence on indoor pollution in dental clinics and the effects on the health of healthcare workers and patients. However, air quality in dental clinics has become a concern; bacteria and microorganisms that are considered toxic and released during procedures can lead to dangerous health effects. 6 Most of the pollution inside dental clinics comes from the external environment; therefore, the building location affects air quality, as well as the work activity and the type of natural or mechanical ventilation. 7 Dental staff are exposed to acrylates due to acrylic resin composites and bonding agents used for fillings. These compounds are known to cause contact allergies in dental staff. However, in the 1990s, reports emerged about cases of asthma also caused by methacrylates. The main volatile acrylates used in dentistry are 2-hydroxyethyl methacrylate and methyl methacrylate. 8 It is now known that indoor pollutants cause allergies and asthma, and in the dental sector, these pathologies are on the increase, mainly caused by methyl methacrylate (MMA), 2-hydroxyethyl methacrylate (HEMA), ethylene glycol dimethacrylate (EGDMA), and triethylene glycol dimethacrylate (TEGDMA). In addition, formaldehyde has been classified as a Group 1 human carcinogen by the International Agency for Research on Cancer (IARC). 9 However, there are still gaps in the scientific literature regarding the potential long-term effects of all environmental pollutants on healthcare workers, as well as on the various types of pollutants. Further gaps are found in the regional data available for limits as well, in consideration of climate and exposure to pollution. 10 11

This systematic review aims to investigate the main pollutants present in dental clinics, in order to understand their characteristics and identify their impact on the health of patients and healthcare workers. Ultimately, the aim is to develop strategies to reduce indoor pollution and improve air quality in dental clinics.

## Methods

### Study Characteristics

**Fig. 1:**
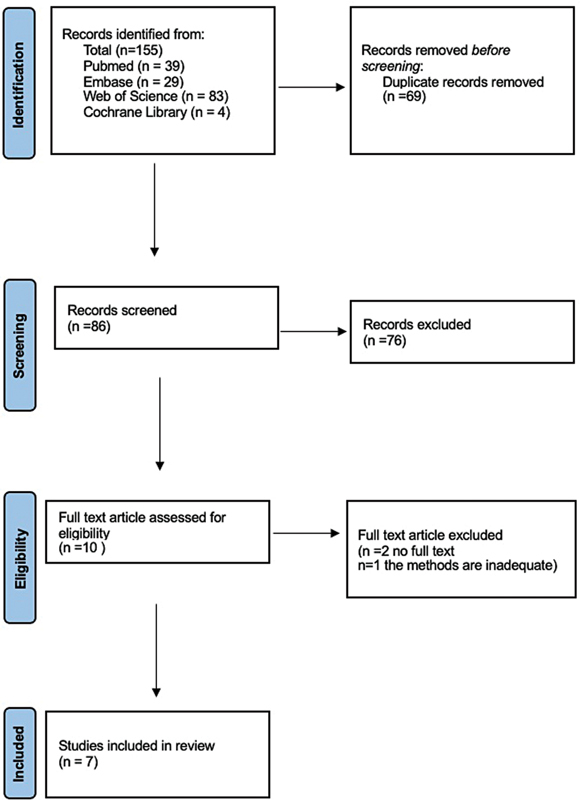
Prisma flow diagram.

The article selection process for this review followed the guidelines provided by the PRISMA flow diagram, as shown in Fig. 1 . A comprehensive search was conducted across PubMed, Embase, Web of Science, and Cochrane Library databases, without time restrictions, resulting in a total of 155 scientific articles. To eliminate duplicates, the references of the identified records were uploaded to the digital tool Rayyan ( http://rayyan.qcri.org , accessed on 11/02/2025). 11 After duplicates removal, 86 unique papers remained for further analysis. The titles of these articles were manually reviewed to include relevant references related to the presence of indoor pollutants in the air of dental clinics. Following this screening process, 10 studies were identified as relevant to the topic of the systematic review. A final selection of seven scientific papers was made because two articles lacked the full text, and the third one was considered inadequate and incomplete in the “Materials and Methods” section.

### Search Strategy

This systematic review adhered to the guidelines employed by the PICO(S) approach (Patient or Population, Intervention, Control or Comparison, Outcome, and Study types). Extensive literature research was conducted using the databases PubMed (Medline), Embase, Web of Science, and Cochrane Library, focusing on the presence of indoor pollutants in dental clinics and their potential effects on operators and patients. 11 The search **strategy used different keywords** , involving “Indoor air quality in a dentistry clinic” or “Indoor air quality in a dental office” OR “Indoor air pollutants in a dental office” OR “Indoor air pollutants in a dental clinic.” The PICO question was expressed as follows: P-population: Air quality inside the clinic/dental practice; I-intervention: actions taken by dentists to improve air quality; C-comparison: no intervention or standard care; and O-outcome: An evaluation of potential strategies to reduce particulate pollution in dental clinics. Articles in languages other than English were not eligible. There was no time restriction for the publication dates of the source documents. As it is considered a systematic review of in vitro studies, the study cannot be registered on Prospero.

The research hypothesis was:

How many studies have been published on air quality in dental clinics?
What types of studies have been published?
What are the main particles in a dental office?
Is it possible to consider a relationship between oral health outcomes and air pollutants in the dental practice?
Are there reference levels for indoor pollutants?

### Inclusion and Exclusion Criteria

Articles were considered appropriate if they met the following inclusion criteria:

1. Articles with a main topic regarding the correlation between indoor air pollution in dental clinics and their impact on oral health.
2. Studies performed in vivo.
3. Full-text paper in English.
4. Retrospective study, case–control study, and cross-sectional study were included.

Articles that did not show the above information were excluded from the review.

Research letters and conference proceedings were excluded.

### Data Extraction and Following Analysis

All the relevant data were extracted, including the author's name, publication year, type of study, pollutants measured, type of dental clinic, and impact of oral health. Specifically, a data abstraction sheet was used, and the accuracy of the information was verified by G. Tetè and M.S. (with the agreement of all the authors).

## Results

### How Many Studies Have Been Published on Air Quality in Dental Clinics?

Seven experimental studies investigating various air pollutants and their association with health-related conditions in dental environments were included. Specifically, measurements focused on volatile organic compounds (VOCs), particulate matter (PM10, PM2.5), nitrogen oxides (NOX), and sulfur dioxide (SO _2_ ), carbon dioxide (CO _2_ ), and temperature levels in a university dental clinic. Due to the heterogeneity of the studies and in consideration of the different type of molecules, different types of clinical facilities, different measurement methods, and different traditional and/or technological ventilation methods used in dental departments, it was not possible to perform a meta-analysis.

### What Types of Studies Have Been Published?

The main results are presented in Table 1 , summarizing the key findings of the seven articles examined. To provide a more comprehensive overview, however, we have created an additional table ( Table 2 ) comparing the type of clinic, the type of occupants, the type of ventilation, the measurement times, the temperature level, and the CO _2_ , PM _2_ . _5_ , and PM _10_ levels. The quality of the studies was assessed using the QUADAS-2 tool, which was developed for in vitro studies through the JBC scientific guidelines (see Tables 3 and 4 ).

**Table 1:** Summarizing the principal results of the study in a dental clinic

Author	Year	Type of indoor pollutant	Type of dental structure	Experimental conditions	Measured variables	Resume of principal results
1. Tzoutzas et al 56	2021	PM2.5, PM10, VOCs and CO _2_	Postgraduate clinic of the Dentistry School of the National and Kapodistrian University of Athens	The clinic where the study was conducted is located on the second floor of the main postgraduate building and occupies a space of 170 m ^2^ (510 m ^3^ ). The clinic operates in two shifts. ** The morning shift is from 08:30 am to 12:25 pm** with approximately **27–35 people** (staff and patients), and the ** noon shift is from 13:30 pm to 16:45 pm** with approximately **16–29 people.** The study period comprised four monitoring periods: **from 29 April to 16 May 2021,** to obtain background data ( **from 29 April to 9 May 2021 when no personnel attended the clinic and from 10 to 16 May 2021 when only security staff visited the clinic** ) and **17–21 May, 24 to 28 May and 31 May to 4** June 2021 when the medical staff worked under different operational patterns of the air purifiers. The existing air conditioning/ventilation equipment of the clinic includes two 6 Kw split units of a cooling capacity, which can cover the cooling load of the ventilation system. The performance of the Aurabeat AG+ NSP-X1 air purifiers (from now on named P1 and P2) installed within the clinic was also evaluated. Four portable Tongdy TSP-18 real-time output of CO _2_ , temperature, and relative humidity (RH) sensors designed for wireless connection via a cloud were placed within the clinic to adequately cover the volume of the room (510 m ^3^ ); each one of them was approximately at a 1-m height from the ground. All instruments were in conformity with the GB/T19001–2016/ISO9001:2015 standard. All data were recorded continuously at 1-min intervals on a 24-h basis and were uploaded onto an online platform with the use of a wireless local area network (WLAN). The sensors performed simultaneous measurements of the temperature and RH levels along with concentrations of CO2, TVOC, PM2.5, and PM10. A 5-wk monitoring protocol was scheduled and IAQ measurements were initiated on 29 April 2021, when no educational activities took place for 15 d, to monitor the threshold concentrations of CO2, PM2.5, PM10, and TVOC (background data)	Concentrations of CO _2_ , TVOC, PM2.5, PM10, temperature, and RH levels	More specifically, >95% of RH measurements were found to be within the range of 30–60% and ∼70% of the temperature was within 23–26 °C. Thus, the thermal conditions within the clinic were adequate. The levels of all investigated air pollutants were low except for TVOC. More specifically, PM10 and PM2.5 presented relatively low concentrations from 2.05 to 72.63 μg/m ^3^ with an average of 6.80 g/m ^3^ and from 1.58 to 71.40 g/m ^3^ (mean: 6.30 g/m ^3^ ), respectively. The levels of CO _2_ were recorded from 375 to 1,407 ppm (average 457 ppm) and concentrations of TVOC ranged from 0.03 to 4.00 mg/m ^3^ with an average value of 0.79 mg/m ^3^ . Fig. 4 shows the concentrations of CO _2_ in the clinic. These results were in compliance with the study of Meyer. 12 In the latter study, the researchers reported that the CO _2_ concentration levels in a dentistry clinic are associated with the number of occupants and the ventilation conditions, which can reach the highest values when the maximum number of people occupy the clinic and insufficient air renewal is noted. The use of mechanical ventilation during all working shifts played a key role in the results, retaining good IAQ levels within the clinic. Air purifiers showed a positive impact on IAQ, mainly by reducing the levels of PM2.5 and secondly of TVOC
2. Liu et al 67	2017	CO _2_ , total volatile organic compounds (TVOCs), suspended particulate matter (PM), and bacteria	Dental Department of the Chang Gung Memorial Hospital in Taiwan. The study was a cross-sectional study design. Indoor air quality (IAQ) evaluations were conducted in six locations: pediatric dentistry, craniofacial orthodontic dentistry, periodontal dentistry, and general practice dentistry, instrument washing room, and patient waiting area	The space volumes of PEDI, PERIO, IR, ORTHO, GP, and PWA were 26.51, 39.22, 31.05, 96.59, 232.03, and 78.62 m ^3^ , respectively. During the sampling period, indoor air was conditioned but not heated. This study was performed from July to August in 2016. Indoor air quality parameters of six locations in the dental department were monitored for 9 h (8 am to 5 pm ) per day for 3 d. Bacterial samples were collected twice a day (in the morning and in the afternoon) for 3 d. The air sampling instruments were placed approximately 1.5 m from the dental unit to avoid interrupting dental treatment, and as close to the center of the sampling area as possible. All instruments were positioned 1 m above the floor to simulate the seated breathing zone of healthcare workers	The evaluated air quality indices included the air temperature, RH, and concentrations of TVOCs, CO _2_ , suspended PM, and bacteria. The air temperature, RH, and CO _2_ concentration were determined every minute using a digital psychrometer (TSI, Inc., Shoreview, MN, USA). The PM levels were measured every 6 s using a portable dust monitor with 31 size channels measuring the size range between 0.25 and 32 μm (model 1.110; Grimm Labortechnik Ltd., Ainring, Germany). The level of TVOCs was determined every minute by a hand-carried detector (ppb RAE 30000, USA). Bacterial concentrations were assessed using Andersen one-stage viable impactors (N6; Andersen Samplers, Atlanta, Georgia) with 20 mL of tryptic soy agar at an airflow rate of 28.3 L/min for 3 min	The largest and smallest numbers of people were observed in ORTHO ( *N* = 105) and in PERIO ( *N* = 66), respectively. The highest temperature was recorded in PEDI (24.5°C), while the lowest was recorded in ORTHO (20.43 °C). The temperature differences in the six locations are statistically significant ( *p* < 0.001). The IR had the highest RH value (70.59%), while PERIO had the lowest RH value (58.11%). Differences in the RH values in the six locations were statistically significant ( *p* < 0.001). The measured CO _2_ concentration in six regions ranged from 491.73 to 653.65 ppm, with the maximum occurring in PERIO and the minimum occurring in IR. A significant difference ( *p* < 0.001) was found in the CO _2_ concentrations for the six locations, but all values were within the TEPA indoor air quality standard of 1,000 ppm. The average TVOCs concentrations found in the six sampling locations all exceeded TEPA indoor air quality standards of 560 ppb/h. The highest concentration occurred in GP (1,373.99 ppb) and the lowest occurred in the IR (674.56 ppb). The TVOC concentrations in the six locations were significantly different ( *p* < 0.001). The maximum concentration of PM10, PM2.5, and PM1 was found in PERIO, while the minimum concentrations were found in the PWA. Concentrations of PM10, PM2.5, and PM1 had statistically significant differences between the six sampling locations ( *p* < 0.001). Concentrations of PM10, PM2.5, and PM1 had statistically significant differences between the six sampling locations ( *p* < 0.001). However, none of the values exceeded the TEPA standard. The upper limit for the PM10 concentration was 75 μg/m ^3^ for 24-h average concentration, while that for PM2.5 was 35 μg/m ^3^ for 24-h average concentration. The median concentrations of airborne bacteria in PERIO (773.01 CFU/m ^3^ ), GP (307.56 CFU/m ^3^ ), IR (619.63 CFU/m ^3^ ), and PWA (1,299.25 CFU/m ^3^ ) were significantly higher than those in ORTHO (84.48 CFU/m ^3^ ) and PEDI (247.97 CFU/m ^3^ ). Also, a significant difference in the airborne bacterial concentration was found between PWA and IR ( *p* = 0.025). In this study, the bacterial concentrations were generally lower than the TEPA indoor air quality standards, which set an upper limit of 1,500 CFU/m ^3^ . Only two specimens exceeded the 1,500 CFU/m ^3^ standard, both during afternoon sessions, one in GP (4,058.67 CFU/m ^3^ ) and one in the PWA (2,551.39 CFU/m ^3^ ) (data not shown). In the morning and afternoon sessions in PEDI and ORTHO, bacterial concentrations were below 500 CFU/m ^3^ . Temperature had a significant negative correlation with RH ( *r* = − 0.463, *p* < 0.01) and TVOCs ( *r* = − 0.16, *p* < 0.05), while RH had a significant negative correlation with CO _2_ ( *r* = − 0.550, *p* < 0.01) and TVOC ( *r* = − 0.172, *p* < 0.05) concentrations ( Table 2 ). In addition, CO _2_ concentration was positively correlated with the concentration of TVOCs ( *r* = 0.377, *p* < 0.01), PM10 ( *r* = 0.28, *p* < 0.01), and PM2.5 ( *r* = 0.16, *p* < 0.05). PM10 had significant positive correlations with PM2.5 ( *r* = 0.827, *p* < 0.01) and PM1 ( *r* = 0.739, *p* < 0.01)
3. Santarsiero et al 70	2011	Air VOCs concentration levels in a work setting	The emergency ward of the dental hospital Ospedale Odontoiatrico George Eastman of Rome was chosen for the investigation. Being a dental facility that operates 24 h a day, it treats ∼38,000 patients per year	The hospital is in an urban area with high air pollution levels, which allows for a strict paired-sample comparison of outdoor/indoor VOC concentrations. It is well known that indoor and outdoor environments are in close contact and undergo significant air exchanges, and the presence of external sources (and sinks) is crucially important for detecting fluxes (Santarsiero et al. 2009). The dental emergency ward is open 24 hour a day. By protocol, only the following procedures are allowed: - Procedures necessary to relieve pain, or eliminate acute infection—e.g., starting root canal treatment on infected teeth, draining abscesses and infected areas; - Procedures involving application of medications, amalgam removal, composite removal, temporary fillings for fractured teeth. The materials and chemical agents used during the course of the dental clinical procedures are: Eugenol (temporary cement), Carbocaine, M-cresol, formalin and glutaraldehyde are ingredients of substances sometimes used as root canal antiseptics and chlorhexidine. The products used for disinfecting medical instruments and dental equipment surfaces contain o-phenylphenol, benzoyl-p-chlorophenol, and N-alkyl-N-benzyl-N,N-dimethylammonium chloride as active ingredients. The Radiello passive sampler (code no. 165) for aldehydes and the Radiello passive sampler (code no. 130) for BTEX and MMA were used (Santarsiero et al. 2009). Six passive samplers were simultaneously placed as follows: (1) Two samplers, one for BTEX and MMA and the other for aldehydes, were set aside at ∼1.70 m height in the breathing zone and at a distance of ∼1.0 m from the dental chair (PIN1—see Fig. 1 —dental chair partitioning 1). (2) Two samplers, one for BTEX and MMA and the other for aldehydes, were set aside at ∼1.70 m height in the breathing zone and at a distance of ∼1.0 m from the dental chair. Two samplers, one for BTEX and MMA and the other for aldehydes, were set aside at the outdoor window of a separate room. The samplers were left for 7 consecutive days during which occupants (dental staff, dentists, etc.) were asked to ignore the existence of the samplers. The in-field sampler preparation took place by installing the collection cartridge that is housed in a sealed glass tube before sampling into the diffusive body that was screwed into the supporting plate. The samplers were retrieved after 7 days' exposure. The cartridge of each sampler was removed and immediately housed again in the sealed glass tube, transported to the laboratory, and kept at 48 °C until VOC analysis. BTEX and MMA were identified and determined on the basis of retention times and confirmed by gas chromatography-mass spectrometry (GC6890 HP-MS 5973 HP) analyses. The identification and determination of individual carbonyls were performed by reverse-phase HPLC (high-performance liquid chromatography). Computations were performed by means of the software SAS for personal computers. The whole dataset consists of 45 variables. To investigate both the presence and nature of indoor/outdoor air exchange and the indoor sources deriving from the dental activity, extracted component scores (i.e., TOTVOCFACT#, BTEXINFACT#, BTEXOUTFACT#, CARBINFACT#, CARBOUTFACT#, and PATOLFACT#) of each sub-set data, plus DRILLing and TOT_PATIENTS were then merged in a matrix and each other correlated. The mutual correlations between treated pathologies, indoor and outdoor pollution components, as well as the number of total patients (TOT_PATIENTS) referred to the dental setting, and the number of drillings (DRILLing), were computed by means of Pearson's correlation coefficients. The partial correlation method was used to grasp hidden correlations—i.e., correlations masked by the effect of other variables—between pollution and pathological variables. Correlation results were then assessed by means of multiple regression analysis, and a model, able to predict the pathologies components by means of the indoor/outdoor pollution	The monitoring consists of the simultaneous measurement of pollution variables (VOC concentrations relative to two indoor locations [two dental chairs partitioning] and one outdoor location [window]), and pathological variables (types of treated pathologies, number of drillings performed at the two dental chairs partitioning, and number of patients referred to the dental setting).Twelve measurements, each lasting for seven consecutive days, were performed on a monthly basis from November 2007 to October 2008. The following VOCs were selected (10): benzene, toluene, ethylbenzene, and m-o-p-xylenes (BTEX), methyl methacrylate (MMA), and eight carbonyls (which are the following aldehydes: formaldehyde, acetaldehyde, propionaldehyde, benzaldehyde, n-butyraldehyde, valeraldehyde, isovaleraldehyde, and glutaraldehyde)	MMA was never detected (for an analytical detection limit of 0.01 mg/m ^3^ ) in outdoor air. Glutaraldhyde was never detected (for an analytical detection limit of 0.01 mg/m ^3^ ) both in indoor and outdoor air. For each individual volatile organic compound, the obtained concentration was below occupational exposure limits set by relevant institutions such as the National Institute for Occupational Safety and Health (NIOSH), the Occupational Safety and Health Administration (OSHA), American Conference of Industrial Hygienists (ACGIH), etc. MMA is originated from a specific indoor source different from the sources of the other compounds. This is a proof of the ‘‘non-use’' of MMA in the emergency ward, in which, by protocol, dental procedures, entailing the use of such a substance, are not allowed. MMA is used in the preparation of various dental products (Marquardt et al. 2009) only in laboratories of dental technicians that are located on another floor of the building. Despite the relatively low concentrations of investigated compounds, well below the occupational exposure limits, it has been demonstrated that formaldehyde, acetaldehyde, propionaldehyde, isovaleraldehyde, and valeraldehyde effectively come from the dentistry activity. MMA was never detected outside, but inside it was always detected, confirming its origin from a specific indoor source as derived by the presence of a factor upon which MMA is specifically loaded independently by the other pollution sources (see Factor 2 of indoor BTEX and MMA sub-set data factor pattern). By protocol, the investigated dental emergency ward only provides for the removal of the composite material. Thus, in our case, the presence of MMA may be attributed to the flow of contaminated air from one ward to another through open doors. This is a proof of the ‘‘non-use’' of MMA in the emergency ward, in which, by protocol, dental procedures, entailing the use of such a substance, are not allowed. The combined use of PCA on the sub-sets of data and the correlation assessment, by means of multiple regression analysis and partial correlations, allowed us to obtain a highly statistically significant linear model linking two different observed pollution factors to the main pathology factor, which allows for a causative model of pollution to be sketched
4. Helmis et al 60	2007	COV total, CO _2_ , il PM10, PM2,5, l'Nox and l'SO _2_	Dentistry Clinic, Dentistry Faculty, of the Athens University, with respect to chemical pollutants, and identify the indoor sources associated with dental activities, which consist of two individual 5-floor buildings (the Undergraduate Studies Building and the Postgraduate Studies Building) connected by an internal corridors	Before the main experiment, preliminary measurements of TVOCs, CO _2_ , and particulate matter concentrations were performed in several areas of the Dentistry Faculty. From the results obtained, the Total Treatment Clinic, on the third floor of the Undergraduate Studies Building, was selected according to its characteristics and high pollution levels. The clinic has an area of 290 m ^2^ and operates in two shifts (08:00–12:30 and 13:00–17:00) with 70–100 occupants in every shift. It is naturally (not mechanically) ventilated. Heating is achieved by central heating radiators and air conditioners (A/Cs), which were rarely used. In this room, the pollutants TVOCs, PM10, PM2.5, NO, NO _2_ , SO _2_ , and CO _2_ were monitored during the period from the 3rd December 2004 to the 3rd March 2005, during both working days and weekends, with various instruments	In this room, the pollutants TVOCs, PM10, PM2.5, NO, NO _2_ , SO _2_ , and CO _2_ were monitored during the period from the 3rd December 2004 to the 3rd March 2005, during both working days and weekends, using the following instrumentation: • Portable instrumentation—two indoor air quality monitors (IAQRAE and ppbRAE of RAE systems) for TVOCs measurements (resolution: 10 and 1 ppb, respectively, accuracy: 10%) and one monitor (IAQRAE of RAE systems) CO _2_ measurements were employed. The TVOCs and CO2 concentrations refer to 1-h mean values, derived from 1-min continuous measurements. The measured values of TVOCs are isobutylene equivalent and conversion from ppb to μg/m ^3^ has been done by multiplying the measured value by the factor 2.3, according to Alevantis and Xenaki-Petreas (1996). The IAQRAE system also provides measurements of temperature and relative humidity, as 1 h mean values • Automated Horiba analyzers measuring NO, NO _2_ , SO _2_ , interfaced to a data logger giving 10-min average values. The NOx analyzer uses a semiconductor sensor and the SO _2_ analyzer uses an optical system. The principle of operation is the chemiluminescence and UV fluorescence for the NOx and SO _2_ analyzers, respectively. The lowest detection limits (LDL) were 0.98, 0.61, and 1.31 μg/m ^3^ , respectively. • Particle samplers measuring PM10 and PM2.5, giving mean concentrations calculated gravimetrically (weighing instrument KERN 770, accuracy 0.01 mg) from pre-set sampling periods (24, 10, and 14 h) (Model 200 Personal Environmental Monitors (PEM) and SKC Universal DELUXE sampling air pumps of 2 L/min). • Outdoor concentrations of PM10 and meteorological data were collected from the air pollution monitoring station operated by the Ministry of Environment (Goudi). The station was located at a distance of 50 m to the south of the Dentistry Faculty. From the meteorological data collected from the station, it was evident that during 52% of the total experimental time, the Dentistry Faculty was downwind of the station, and 48% upwind. During the whole experimental period, a logbook was kept recording all the activities taking place in the clinic, including the number, the location, and the duration of the open windows, the number of students and personnel occupying the room and the nature of their work, the materials used, as well as the cleaning processes and hours. Outdoor CO _2_ concentrations were frequently monitored during the experiment and ranged on average at 1,170 mg/m. Indoor emission rate of CO _2_ was considered mainly due to human respiration and was taken to be 589 mg/min CO _2_ per person. Intensive TVOCs and PM measurements were performed, at two different locations of the room, in the central part (location K) and in the northern part (location B) simultaneously, during the period of 17–25 February 2005. The indoor environmental conditions were examined by applying the CFD model PHOENICS for the 19th, 23rd, 24th, and 25th February 2005, and the indoor production of particulate matter was assessed by employing indoor air quality model MIAQ for the 23rd February. The following instruments were used to measure CO _2_ and temperatures: DANTEC Flow Masters (type 54N60) for spot mean air velocity, temperature, and turbulence intensity measurements of 1-min sets (accuracy 0.1 cm/s, 0.1 °C and 1%, for velocity, temperature, and turbulence intensity, respectively). Infrared thermometer (Cole-Palmer, 08406) for surface temperature measurements of the indoor materials. IAQRAE for CO _2_ measurements	The indoor air quality of a dentistry clinic was studied both experimentally and theoretically. It was found that the indoor air quality with respect to TVOCs, CO _2_ , and PM was critical in the dentistry clinic due to the use of specific substances for dental operations, cleaning processes, and the high number of occupants in the room. The commonly used natural ventilation schemes cannot offer sufficient air renewal throughout the indoor space of the clinic. This causes accumulation and trapping of air pollutants in certain areas of the room, especially in the northern part, and, thus, in the formation of localized high pollution spots, isolated from the general flow. Depending on the occupancy and the ventilation conditions, they reached the highest values when the maximum number of people and insufficient air renewal occurred. Acceptable CO _2_ levels for comfort conditions were only observed at a relatively small indoor area close to the open door. Differently oriented open windows were not found to considerably affect the indoor conditions, while the double cross-ventilation scheme was the most effective. The significantly high levels of TVOCs concentrations recorded in the clinic were attributed to the use of acrylic substances and dental materials. Exceptionally high values were associated with the use of the dental substance Kalocryl. The detergents products used for the decontamination of the working posts also contributed to the enhancement of TVOCs, leading to peaks at the beginning and the end of the shifts and increased background values well above the limits set for the indoor environment. The PM concentration levels were significantly high during the working days, far exceeding the EU limit for the outdoor environment. The emission and diffusion of the particles in the clinic can be attributed to handpiece operation, the trimming of the models, the shaping of the temporaries, the material agitation, and the materials mixing using the hand mode. Specifically, indoor values were much higher than outdoors by a factor of 12. The estimation of the relative contribution of the indoor particle sources, by using the MIAQ model, gave an average of ∼2,400 μg/min for PM2.5 and a total of ∼5,000 μg/min for PM10, directly emitted in the indoor environment, severely affecting indoor air quality. Regarding NO, NO _2_ , and SO _2_ , no indoor sources were present; thus, concentrations were found to be low and followed the outdoor low values and variations
5. Helmis 78	2008	TVOCs, CO2, PM10, PM2.5, SO _2_ and NOX were measured and indoor sources associated with dental activities were identified	Two clinics of the Athens University Dentistry Faculty were part of the study. Both clinics have the same surface area of 150 m ^2^ but different occupancy and operation programs, they are naturally ventilated and heating is achieved by central heating radiators and air conditioners (A/Cs), which are also used for cooling	The Endodontics, Periodontics and Operative Dentistry (EPOD) Clinic is located on the second floor of the building and operates during the hours 08:00–12:30 and 13:00–17:00 LST (local standard time) with a maximum of 30 occupants in every shift. The Prosthodontics (PRO) Clinic, on the fourth floor, operates in one shift from 09:00 to 16:00 LST with a maximum of 20 occupants. In both clinics, continuous measurements of CO2, TVOCs, PM10 (particles with a diameter smaller than 10 μm) and PM2.5 (particles with a diameter smaller than 2.5 μm) were performed, with the same instrumentation. The experimental period was 4 March to 18 April for EPOD Clinic and 19 April to 23 May 2005 for the PRO Clinic. Furthermore, during the period 4–28 March 2005, NOx and SO _2_ concentrations were recorded simultaneously inside and outside the EPOD Clinic, to compare the pollution levels. The outdoor CO _2_ concentrations were monitored during the experiment (ranged on average at 1,170 μg/m ^3^ ) and the indoor emission rate of CO _2_ was considered mainly due to human respiration and it was taken to be 0.3 L/min CO _2_ per person 10. To quantify the amount of TVOCs emitted from different dental materials, additional TVOCs measurements were performed in a nearby dental room of the PRO clinic (area of 14 m ^2^ ), using the portable air quality monitor ppbRAE of RAE systems with a sampling frequency of 5 s. Before the measurement commenced for each material, the background TVOCs levels in the room were measured; while during the measurements, the door and windows were closed, and at the end of each set of measurement, the windows were opened to ventilate the room and thus continue with the next dental material	To quantify the ventilation prevailing in each clinic, the air change rates (ACH) were calculated with a methodology based on the solution of the mass-balance equation for the CO2 concentrations, considering negligible concentration gradients and deposition. The instruments used for the indoor measurements were: • Automated Horiba analyzers measuring NO, NO _2_ , SO _2_ , interfaced to a data logger giving 10-min average values, with 1-s sampling frequency and 1-min data-logging interval. The lowest detection limits (LDL) were 0.98, 0.61, and 1.31 μg/m ^3^ , respectively. Three Teflon valves (three-port) alternated sampling between the indoor and the outdoor air on a 15-min cycle for the EPOD Clinic measurements. • Portable instrumentation—Two indoor air quality monitors (IAQRAE and ppbRAE of RAE systems) for TVOCs measurements (resolution: 10 and 1 ppb, respectively, accuracy: 10%, LDL: 20 ppb) and one monitor (IAQRAE of RAE systems) for CO _2_ measurements were employed at all measurement sites. The TVOCs and CO _2_ concentrations refer to 1-h mean values, derived from 1–min continuous measurements. • Particle samplers measuring PM10 and PM2.5 (PEM systems—SKC Universal DELUXE pumps), giving mean concentrations calculated gravimetrically (weighing instrument KERN 770, accuracy 0.01 mg) for pre-set sampling periods (24, 10, and 14 h). The materials selected for monitoring was the typical substance mostly used in dental clinics: • Eugenol, local antiseptic/analgesic • Copalite (Harry Bosworth), cavity varnish • Scotchbond One (3M/ESPE), dental adhesive • Bacillol (Bode), surface disinfectant • Kalocryl (Speiko), acrylic substance. For each substance, three sets of measurements were performed with a 5-min time period for each set: • At zero distance from the substance (position A) • At a distance of 1.5 m (position B) • At a distance of 4 m (position C)	For each dental substance that are presented, extremely high TVOCs emissions were revealed compared with the threshold limit of 300 μg/m ^3^ , set for the indoor environment according to the international bibliography 14–16, while even the TVOCs background concentrations in the room during the measurements were 800 μg/m ^3^ . The TVOCs emitted from the materials Eugenol, Copalite, and Scotchbond reached values of the order of 2,000, 7,000, and 12,000 μg/m ^3^ , respectively, close to the source and had minor effects at a short distance from the source. On the other hand, TVOCs emissions from Kalocryl and Bacillol close to the source reached extremely high values 120,000 and 40,000 μg/m ^3^ , respectively, but they also affected the air at short and long distances from the source. Bacillol is a material frequently used by dental personnel in large quantities; thus, to reduce the relevant emissions, it is suggested replacing this material with alternative products emitting lower values of VOCs. Regarding the indoor air quality assessment, increased CO _2_ concentrations were observed during working hours, frequently exceeding the international limit, mainly produced by exhalation procedures, being related to the presence of students, patients and medical staff. The average concentration values of TVOCs in both clinics exceeded by far the recommended limit, while the ranges of the maximum recorded hourly values were 1,000–3,650 μg/m ^3^ and 900–14,900 μg/m ^3^ in the EPOD and PRO Clinics, respectively. Even the background concentration average levels (480 and 400 μg/m ^3^ in EPOD and PRO) were higher than the limit. The TVOCs concentrations were influenced mainly by the dental activities, the dental materials used and the ventilation conditions in each clinic, leading to different concentration averages and hourly levels. It was found that Kalocryl and Bacillol were the main TVOCs sources that led to significantly high levels of concentrations, while detergent cleaning products were responsible for high values at the beginning and end of each shift. The estimated exposure of the dental personnel, students, and patients during working hours gave considerably high values for both clinics, exceeding by far the limit value for 8- and 4-h exposure, respectively. It was also found that the PM concentration levels were substantially high in both clinics. The intense indoor sources seem to play the most important role in the formation of the high indoor PM levels, rather than the penetration of outdoor pollution. Thus, in the majority of the days, the PM10 indoor values exceeded the limit in both clinics, while PM2.5 values exceeded this limit during 25% of the days in the EPOD Clinic and only once in the PRO Clinic. On the contrary, the concentrations of the gaseous pollutants SO _2_ , NO, and NO _2_ were characterized by low values, in the absence of significant indoor sources and the outdoor environment seemed to contribute to these the most
6. Hong et al 62	2015	levels of volatile organic compounds (VOCs) and particulate matter (PM)	Dental clinic in southern Taiwan and dental care personnel's health risks associated. The clinic has 11 separate office spaces for dental treatments and procedures	Air quality monitoring took place in the Prosthesis and Filling Process department. The clinic generally used ∼21 different dental materials. It has a commonly used recirculating ventilation system, including an air handling and fan coil unit. The ventilation system captured air through filters and vented the filtered air back into the room. The size of the office is 126.77 m ^2^ , with the height of the ceiling at 2.21 m and the size of an entrance door at 3.1059 m ^2^ [(2.03 m (H) £ 1.53 m (w)] ( Fig. 1 ). The system has 2 filtration units with a minimum air exchange number of 10. The clinic is located on the second floor of the building and operates during the business hours of 08:00–12:00 and 14:00–17:00 Monday through Friday, and 8:00–12:00 on Saturday. Six sampling sites in the clinic were selected based on the ventilation system in the clinic and dental activities. Air samplers were set up at a height of 2.0 m from the floor. Two sites (Sampling 1 and Sampling 2) served as the comparison group and four sites (Sampling 3, 4, 5, and 6) were used as the study group to allow for an evaluation of the distribution of tested pollutants in this clinic. Sampling 1 served as the comparison group and was located at the exhaust area of the ventilation system. The samples from this site were compared with those from the study group to assess whether ventilation could improve the air quality of the dental clinic. Sampling 2 also served as a comparison group and was located at the top of the entrance door to the clinic. The samples from this site were compared with those from the studied group to assess whether natural air exchange could reduce air pollutants in the clinic. Sampling 3 was located in the corner of the room. Sampling 4 was located in the corner of two treatment stations to assess whether dental treatment procedures changed the distribution of pollutants. Also, Sampling 6 was located in the corner of two other treatment stations. Sampling 5 was located in the middle of the room	An automatic, continuous sampling system and a multi-gas monitor were employed to quantify the air pollutants, along with environmental comfort factors, including temperature, CO2, and relative humidity at six sampling sites in the clinic over eight days. Both non-carcinogenic and carcinogenic VOC compounds were assessed based on the US Environmental Protection Agency's Principles of Health Risk Assessment in terms of whether those indoor air pollutants increased health risks for the full-time dental care professionals at the clinic. An air monitoring occurred continuously for 24 h/d, which covers the activities during business hours (08:00–12:00 and 14:00–17:00, Monday–Friday; 08:00–12:00, Saturday) and non-business hours (12:00–14:00 and 17:00–08:00). A multi-gas monitor (model 1302, Bruel & Kjaer, Nærum, Denmark) and a multi-point sampler (model 1303, Bruel & Kjaer, Nærum, Denmark) with photo-acoustic infrared spectroscopy were used to collect VOC levels at the six selected sampling sites 24 h/d during an 8-d period. The detected VOC levels were automatically and continuously transferred to a computer to record the readings at every 1-min interval. The 1-min measurements were used to yield 1-hour mean values of VOCs. Samples were collected from the locations where high VOC levels to detect specific compounds of VOCs by using the multi-gas monitor. The TO-15 samples were concentrated by an Entech 7100 and analyzed by gas chromatography/mass spectrometry. 12 The wavelength of 3.4 mm with a detection limit of 10 ppb was used for the detection of butane, ethyl benzene, ethanol, formic acid, methyl ethyl ketone, naphtha, petroleum distillates, toluene, turpentine, and xylene, whereas the wavelength of 3.6 mm with a detection limit of 36 ppb was used for acetaldehyde, acrolein, formaldehyde, N-methyl-methanamine, and triethylamine. A particulate matter collector (Grimm Technologies, Inc., Douglasville, GA, USA) was used to detect PM10 and was built into an automatic, continuous sampling system (ASS), which included a flow meter with a pump. A CO _2_ monitor, a wind speed indicator, and a thermometer were built into the ASS system to measure CO _2_ , temperature, humidity, wind velocity, and turbulence intensity, which were used to determine a level of environmental comfort in the dental clinic. In addition, CO _2_ levels could be used to assess the efficiency of the clinic's ventilation system. The normality of the distributions of VOCs and particulate pollutant levels was assessed before statistical analysis. The results are presented as mean § standard deviation for better comparison with relevant literature data. Bivariate analysis [the Mann–Whitney U-test, (Z,P)] was conducted to test any significance between the measurements during business hours and non-business hours. Analysis of variance (ANOVA) was conducted to assess any significant differences among the six sampling locations. Data analysis was performed using SPSS for Windows	The relative humidity, temperature and CO _2_ levels suggested that the acceptable comfort conditions prevailed in the clinic. Increased VOC levels were recorded during the dental office's normal operating hours. Particularly, the two highest levels of VOCs were observed at 11:00 and 16:00, as the number of patients visiting the clinic increased. Also, the average level of VOCs exceeded the Taiwan EPA recommended level and the State of Washington's building standard of 0.3 ppm and 0.5 ppm, respectively. VOC levels gradually increased as the morning operations progressed and decreased during the lunch break. A similar pattern also occurred during the afternoon shift. Materials, including Tempron, Rebaron, and Coltoflax, used in this clinic could contribute to the elevated VOC levels. Comparisons of VOC concentrations from six sampling sites revealed that natural air ventilation from the entrance door and the air ventilation system did not generate enough air circulation to reduce VOC concentrations to the EPA's guideline value of 0.3 ppm. The Taiwan EPA guideline is based on the total concentration of 12 VOCs: benzene, carbon tetrachloride, chloroform, 1,2- dichlorobenzene, 1,4-dichlorobenzene, dichloromethane, ethyl benzene, styrene, tetrachloroethylene, trichloroethylene, toluene, and xylene. The average concentration of VOCs detected in the clinic (5.44 ppm) could possibly cause irritation and discomfort to occupants, depending on other air and building parameters. It is important to note that the VOC concentration during non-business hours was 1.3 times lower than during the business hours period, but still exceeded the Taiwan EPA guideline level. Unexpected high concentrations of VOCs were recorded during the weekends when no dental activities took place in the dental clinic. The highest single reading of VOCs (6.99 § 3.35 ppm) was recorded on Saturday afternoon. We speculated that renovation work on the third floor could have contributed to such high VOC levels, since the clinic was located right at the entrance to the third-floor stairway where the renovation took place. The renovation mainly involved the replacement of the floor carpet, which contained VOC-emitting materials. Also, the air ventilation system could not reduce such high VOC levels efficiently from the renovation during the weekends, which could have allowed VOCs to linger and remain elevated during the weekend cycle. The ventilation capacity was not sufficient to avoid the buildup of VOCs and PM10. Clinics and buildings in Taiwan commonly use a recirculating ventilation and air conditioning system that includes minimum dilution rates with outdoor air. That design differs from most commercial and office building environments, which use a mix of outdoor air. Recently, some systems have been introduced for pollutant control (e.g., high-volume evacuation with a local exhaust system). It was alarming that the detected formaldehyde levels exceeded the World Health Organization's indoor air guideline value of 0.1 mg/m ^3^ and the Taiwan EPA's guideline value of 0.08 mg/m ^3^ for a 1-h sampling measurement. Those guideline values were developed to prevent acute and chronic sensory irritation and cancer at the portal-of-entry sites. During the course of this study, we also detected significantly higher levels of formaldehyde during non-business hours and the weekend, when no activities took place in the clinic. We also observed high VOC levels during these times. A total of 68 different kinds of VOCs were detected with a total concentration of 3.5 ppm. Methyl methacrylate showed up as the highest concentration (2.8 ppm). Certain detected VOCs were possible carcinogens, which included methylene chloride (0.004 ppm), chloroform (0.005 ppm), chloromethylbenzene (0.001 ppm), and benzene (0.013 ppm). Other detected VOCs, which were not carcinogens and had relatively high concentrations, included acetone (0.176 ppm), isobutene (0.047 ppm), n-butane (0.090 ppm), toluene (0.034 ppm), propane (0.055 ppm), and 1,2,4,-trichlorobenzene (0.022 ppm). These compounds may not pose longer-term systemic effects of concern because they yielded hazard indexes of less than 1. PM10 did not yield certain peak levels like VOCs. We observed significantly increased PM10 levels during business hours compared with non-business hours. The increased PM10 levels during the business hours met the Taiwan EPA limit of 150 mg/m ^3^ , but exceeded the US EPA's international limit of 65 mg/m ^3^ . The average PM10 concentration for non-business hours was 29 mg/m ^3^ , which meets both the Taiwan NIOSH's and the US EPA's recommended limits. An elevated level of PM10 in the afternoon shift on Wednesday was recorded
7. Rexhepi et al 16	2021	PM1, PM2,5, PM10, and breathable (<4 microns) total dust during 14 procedures performed with and without the presence of natural ventilation in a dental unit	A dental unit located in an open plan clinic of the Department of Innovative Technologies in Medicine and Dentistry of the University “G. d'Annunzio” of Chieti-Pescara, Italy, was selected for this study	The measurements in the dental office were conducted during a 3-wk period in September 2020. During this period, dental activities were limited to organizing patient flux, following the indications of the Ministry of Health. Dental activities such as professional oral hygiene practices, conservative dental therapy, prosthetic reconstruction, dentoalveolar surgery, and implant surgery were included for the study evaluations. Specifically, restorative treatments were performed using a rubber dam as the method of isolation of the operative field, dental extractions involved tooth sectioning without performing soft tissue incisions and ostectomies, and implant surgery was performed without any bone regeneration intervention. Tooth preparations for fixed prostheses were performed using the high-speed, high-torque handpiece of the unit chair. A real-time monitor was used to measure particulate matter (PM) mass fractions for each particle size every 2 seconds during the procedure analyzed, and the measurement duration was 90 min for each procedure, consisting of continuous slots including leaving the room in a standstill condition with no operation, performing the dental procedure, and cleaning and sanitizing the operating room. Thus, 15,574 values were recorded in each session. Each test lasted an average of 40 min. Each test, therefore, acquired ∼1,112 values. Statistical analysis was performed using SPSS software 11.0 (SPPS Inc, Chicago, IL, USA), and correlated analysis was applied to PM1, PM2.5, PM10, breathable, and total dust. The variables are reported as media, standard error, minimum, and maximum. The statistical analysis was performed by the parametric test, due to the high number of recorded measurements (15,574). The Student's *t* -test was used for unpaired data to compare quantitative variables in the two groups. The statistical significance of the differences between the groups was evaluated at an alpha level of 0.001	Fourteen different dental procedures were performed with the DustTrak DRX Aerosol Monitor Model 8534 (DustTrak TSI Incorporated, Shoreview, MN, USA). 16 This real-time monitor conforms with ISO 21501–4 and is accurate to within 95% with a 5% particle coincidence loss. The sampler software allows data to be analyzed, synthesized, and graphed. The goals of this study were, therefore, to investigate: -The effects of natural ventilation on the reduction of PM in the dental unit -The effectiveness of low-volume suction (40 L/min air) in reducing PM concentrations during dental procedures -The difference in terms of PM between the scaling procedures and the other procedures performed. This discriminant was selected following previous literature and guidelines that described ultrasonic scaling as the dental activity that produces the greatest amount of airborne contamination. Different dental practitioners at the Department of Innovative Technologies in Medicine and Dentistry performed the dental procedures included in the study using the same chair unit. The room's dimensions were 2.8 m × 2.8 m × 3 m (W × L × H). All practitioners used protective equipment during the procedures, including a face mask (filtering facepiece level 2), gloves, a face shield, goggles, and protective outerwear such as disposable gown	The present study demonstrated that the total concentration of PM produced during the dental procedures can be influenced by several factors in clinical daily practice, such as ventilation, type of procedure, or the use of saliva standard ejectors. Our data provide information on the aerodynamic characteristics of PM release, as well as indications that might be followed during daily dental practice. Moreover, an adequate suction system can considerably reduce PM10 aerosol distribution during dental procedures. In fact, saliva standard ejectors showed a good efficacy in reducing the amount of PM10 particles and total dust produced, but the device seemed less efficient in the aspiration of ultrafine particles, with a reduction of 23% of PM1 and of 50% for total dust, with no statistically significant difference. In this context, our results showed that natural ventilation increases the amount of PM if the dental unit door is also kept open during dental procedures, probably due to the aerodynamic mechanism of aerosol diffusion mentioned above. Additionally, for the dental procedures performed, our data showed that without natural ventilation, scaling procedures produced higher PM particle levels compared with other monitored dental activities, in agreement with what was previously reported in the literature. Otherwise, no differences have been proven in the effectiveness of mechanical over manual instrumentation in non-surgical periodontal procedures, and therefore, it is recommended to use manual instruments. Moreover, it was shown, in agreement with our data, that the values of PM1 and PM2.5 did not vary significantly immediately after the initial measurements, since it is assumed that PM10 is heavier and therefore would have precipitated more extensively than PM1 and PM2.5

**Table 2:** Comparing the type of clinic, the type of occupants, the type of ventilation, the measurement times, the temperature level, and the CO _2_ , PM _2_ . _5_ , and PM _10_ levels

Article	RH	Temperature	PM _2,5_	PM _10_	CO _2_	Employees	Type of clinic	Type of ventilation	Time of measurements
1. Tzoutzas et al 56 (2021)	30–60% (95%)	23–26° (70%)	6.80 g/m ^3^	6.30 g/m ^3^	457 ppm/822.604 mg/m ^3^	The clinic operates in two shifts. The morning shift is from 08:30 am to 12:25 pm with ∼27–35 people (staff and patients) and the noon shift is from 13:30 pm to 16:45 pm with ∼16–29 people	Faculty of Dentistry of the National and Kapodistrian University of Athens (Athens, Greece) space of 170 m ^2^ (510 m ^3^ )	The existing air-conditioning/ventilation equipment of the clinic includes two 6 kW split units + Aurabeat AG+ NSP-X1 air purifiers + 2 portable HEPA units	All data were recorded continuously at 1-min intervals on a 24-h basis and were uploaded onto an online platform with the use of a wireless local area network (WLAN). The sensors performed simultaneous measurements of the temperature and RH levels along with concentrations of CO2, TVOC, PM2.5, and PM10
2. Liu et al 67 2017	58,11–70,59%	20.43–24.5°(ORTHO-PERIO)	TVOC 674,56 ppb- 1373,99 ppb	TVOC 674,56 ppb- 1373,99 ppb	491,73 to 653,65 ppm/885.118 mg/m ^3^ to 1,176.576 mg/m ^3^	During the study period, the largest and smallest numbers of people were observed in ORTHO ( *N* = 105) and in PERIO ( *N* = 66), respectively	six locations of the dental department, including pediatric dentistry (PEDI), craniofacial orthodontic dentistry (ORTHO), periodontal dentistry (PERIO), general practice dentistry (GP), the instrument washing room (IR), and the patient waiting area (PWA). The space volumes of PEDI, PERIO, IR, ORTHO, GP, and PWA were 26.51, 39.22, 31.05, 96.59, 232.03, and 78.62 m ^3^	NA	9 h (8 am to 5 pm) per day for 3 d
3. Santarsiero et al 70	2011	NA	NA	NA	NA	Being a dental facility that operates 24 h a day, it treats ∼38,000 patients per year, which ensures the representativeness (Ortolani and Santarsiero 2008) of the studied case for the general issue of dentists and dental staff exposure to air VOCs concentration levels in a work setting	Ospedale Odontoiatrico George Eastman of Rome was chosen for the investigation	NA	Twelve measurements, each lasting for seven consecutive days, were performed on a monthly basis from November 2007 to October 2008
4. Helmis et al 60	2007	23.7°	75 μg/m ^3^	138 μg/m ^3^	1,600 (mg/m ^3^	The clinic has an area of 290 m ^2^ and operates in two shifts (08:00–12:30 and 13:00–17:00) with 70–100 occupants in every shift	Dentistry clinic of the Athens University Dentistry Faculty	Multiple types and combinations of ventilation systems to evaluate the most effective one	approximately 3 mo in a selected dentistry clinic
5. Helmis 78	2008	NA	44 μg/m ^3^	86 μg/m ^3^	1,520 mg/m ^3^	50 occupants	An assessment of the indoor air quality in two clinics of the Athens University Dentistry Faculty was conducted	Mixed ventilation	The experimental period was 4 March to 18 April for EPOD Clinic and 19 April to 23 May 2005 for PRO Clinic. Furthermore, during the period March 4–28, 2005, NOx and SO _2_ concentrations were recorded simultaneously inside and outside the EPOD Clinic, to compare the pollution levels
6. Hong et al 62	2015	23.8°	NA	52 mg/m ^3^	941.9 ppm/1,695.428 mg/m ^3^	NA	A dental clinic in southern Taiwan	It has a commonly used recirculating ventilation system, including an air handling and fan coil unit. The ventilation system captured air through filters and vented the filtered air back into the room	An air monitoring occurred for 24 h a day, which covers the activities during business hours (08:00–12:00 and 14:00–17:00, Monday to Friday; 08:00–12:00, Saturday) and non-business hours (12:00–14:00 and 17:00–08:00). The measurements during business hours were conducted to determine variations in indoor air pollutants related to the activities performed in the dental clinic, while those during non-business hours assessed the background levels of indoor air pollutants. During the sampling period, we recorded all activities, including the number of personnel and clients in the clinic, the location and the duration of time that the doors remained open, the dental procedures in each room and the duration of processing times for each procedure
7. Rexhepi et al 16	2021	NA	35 mg/m ^3^	44 mg/m ^3^	NA	NA	A dental unit located in an open plan clinic of the Department of Innovative Technologies in Medicine and Dentistry of the University “G. d'Annunzio” of Chieti-Pescara, Italy, was selected for this study	Natural ventilation	The measurements in the dental office were conducted during a 3-wk period in September 2020. During this period, dental activities were limited to organizing patient flux, following the indications of the Ministry of Health

**Table 3:** 4 QUADAS-2 tool quality assessment (* = grade of risk)

Author	Patient selection	Index test	Reference standard	Flow and timing
Tzoutzas et al 56	**	**	**	**
Liu et al 67	**	**	**	**
Santarsiero et al 70	**	**	**	**
Helmis et al 60	**	**	**	**
Helmis et al 78	**	**	**	**
Hong et al 62	**	**	**	**
Rexhepi et al 16	**	**	**	**

**Table:** 

Study	Risk of bias				Applicability concerns		
	Patients selection	Index test	Reference standard	Flow and timing	Patients selection	Index test	Reference standard
Tzoutzas et al 56				?			
Liu et al 67				?			
Santarsiero et al 70				?			
Helmis et al 60				?			
Helmis et al 78				?			
Hong et al 62				?			
Rexhepi et al 16				?			

**Table 4:** Updated AQGs compared with those proposed in 2005, also compared with those of the Italian legislation, 43 showing the interim concentration values for each pollutant

Pollutant	Time reference	Interim values, mg/m ^3^				OMS Guidelines 2021	OMS Guidelines 2005	DLgs 155/2010 Italy
		1	2	3	4			
PM _2,5_	Annual	35	25	15	10	5	10	25
	24 h	75	50	37.5	25	15	25	–
PM _10_	Annual	70	50	30	20	15	20	40
	24 h	150	100	75	50	45	50	50
O _3_	Seasonal peak value	100	70	–	–	60	–	–
	24 h	160	120	–	–	100	100	–
NO _2_	Annual	40	30	20	–	10	40	40
	24 h	120	50	–	–	25	–	–
SO _2_	24 h	125	50	–	–	40	20	125
CO	24 h	7 mg/m ^3^	–	–	–	4 mg/m ^3^	–	–

### What Are the Main Particles in a Dental Office?

Most of the articles included in the systematic review deal with PM and VOCs (six out of seven articles), while the others include CO _2_ , NOX, and SO _2_ as well. High concentrations of aerosols can be produced during dental procedures that involve a high-speed drill or a rotary instrument. 12 13 14 Aerosol composition, concentration, and distribution are influenced by factors such as the type of treatment, the size and location of the treatment room, the duration of treatment, patient characteristics, and seasonality. 15 16 Dentists are potentially exposed to various air pollutants during different types of dental procedures. The use of resin materials, particularly in prosthetics, results in the release of microparticles into the air that can have long-term effects on health care operators and patients. 17 In this regard, the World Health Organization (WHO) Working Group on Assessment and Monitoring of Exposure to Indoor Air Pollutants concluded that indoor formaldehyde concentrations > 100 µg ^−3^ are enough to require corrective action based on health effects. 18 Indoor ammonia concentrations were usually less than 20 µg ^−3^ . 19 High ammonia concentrations (up to 210 µg ^−3^ ) have been reported to cause irritation in humans. 20 21 22 23

### Is It Possible to Consider a Relationship between Oral Health Outcomes and Air Pollutants in the Dental Practice?

The role of indoor and outdoor air pollution as a major environmental risk factor for health is now well established, with around 7 million premature deaths per year worldwide, including around 400,000 in Europe. 24 25

**Fig. 2:**
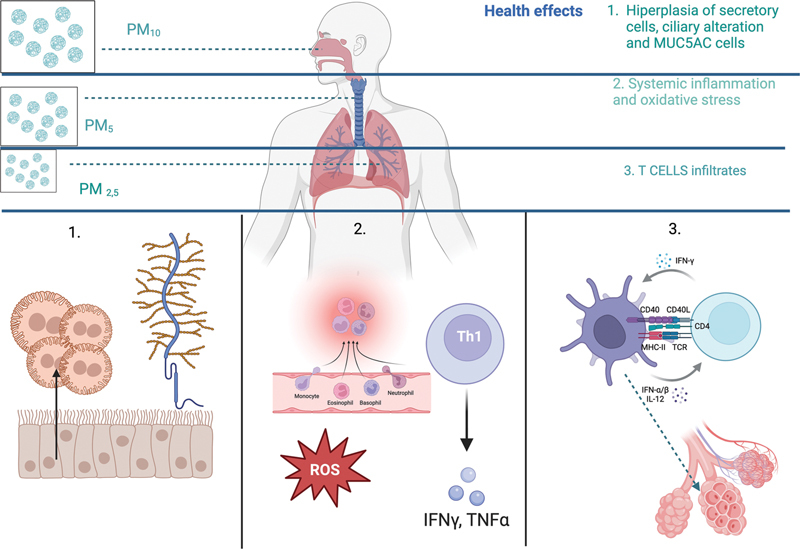
Main health effects and anatomical extent of the particulate.

Recently, the scientific community has become increasingly interested in the quality of indoor air in hospitals and healthcare facilities. 26 Operating rooms, biochemical laboratories, wards, and private offices have been studied where the mixture of pollutants, chemical compounds, microorganisms, and infectious biological agents in the air creates indoor conditions that are dangerous for the health of both patients and health care workers. 27 28 29 Comparison of this type of environment in hospitals with different exposures to different risk factors, with or without air conditioning, has shown the positive effect of ventilation systems in improving indoor air quality, provided these systems are properly operated and well maintained ( Fig. 2 ). 30 31

The prevalence of allergies, asthma, and other respiratory diseases has increased significantly in recent decades. Among other factors, this phenomenon has been linked to the adverse health effects of air pollution, 32 which is often much higher in indoor than outdoors environments. Volatile organic compounds (VOCs) have been implicated as causal agents in asthma and building-related diseases. 33 In the case of dental environments, an increase in occupational respiratory diseases such as asthma caused by VOMs has been observed in dental personnel in recent years. 34 35 Volatile methacrylates such as methyl methacrylate (MMA), 2-hydroxyethyl methacrylate (HEMA), ethylene glycol dimethacrylate (EGDMA), and triethylene glycol dimethacrylate (TEGDMA) have been analyzed in the air on a dental chair during dental filling. 8 36 Less information is available on the levels of other volatile organic compounds, such as benzene, toluene, ethylbenzene, and xylenes (BTEX), which are considered markers of VOC exposure, and some aldehydes, such as formaldehyde, which has been classified as a Group 1 human carcinogen by the International Agency for Research on Cancer (IARC). The currently available data on VOC levels. 37 38 Dental environments are mostly representative of the specific dental environment studied, with a very low power of generalization. 8 19 20 21 22 23 24 25 26 27 28 29 30 31 32 33 34 35 36 37 38 39 Gas phase pollutants present in dental offices include gases such as nitrous oxide (NO), volatile organic compounds (VOCs), and mercury vapor. NO is emitted during anesthetic dentistry, with well-known effects on health during prolonged clinical dentistry, but this phenomenon is reduced using abatement systems. 40 41 ICOVs emitted in dental offices include methanol, methyl acrylate, methyl methacrylate, and isobutyl methacrylate associated with resins and solvents. 11 The manufacture of crowns and bridges, acrylic frameworks, and dentures is a major source of alloys, porcelain, mercury, and methyl methacrylate, which are the most common materials found in dental laboratories. 42 These pollutants have a significant impact on patients' health, who are exposed to high concentrations for short periods of time, but the effects are limited to irritation of the eyes, nose, and throat, and headaches, nausea, or dizziness. However, for students and doctors who spend most of their time in the clinic, long-term exposure can cause respiratory diseases, dermatological problems, allergies, and neurotoxicity. 43 However, the status of air quality with respect to gaseous and particulate pollutants in dental clinics has not been thoroughly investigated. 44

### Are There Reference Levels for Indoor Pollutants?

Table 4 summarizes the updated AQGs compared to those proposed in 2005, also compared to those of the Italian legislation, 45 and shows the interim concentration values for each pollutant. In fact, these are the environmental levels defined to help decision-makers, especially in the most polluted areas of the world, to adopt stricter policies in order to implement a realistic pollution reduction path. The reductions in the guideline values are significant for all pollutants, particularly for the annual values of PM and NO, for which a guideline value is also introduced for the daily average, which did not exist previously. 46 47 48 Only for SO do the new recommendations suggest a higher 24-hour value than the previous one, based on new assessments of short-term effects. 49 50 Some short-term exposure guideline values remain unchanged for CO, NO, and SO ( Table 4 ).

Certainly, the discrepancy between the current legal values and the levels of the WHO AQGs must stimulate the identification and adoption of ambitious, structural, synergistic, integrated, and coherent actions in the various sectors, from industry to civil society, and at all regional, national, and European levels, to achieve the continuous reduction targets in the near future. 51 Given the importance of this issue at a global level, we have made a table of the values found in different dental facilities, reported in the articles included, and compared them with the normative reference values ( Table 5 ).

**Table 5:** Values found in different dental facilities, reported in the articles included, and compared them with the normative reference values

Articles	Type of dental clinic	Pollutants and results (medium levels)	OMS guidelines 2001	OMS guidelines 2005	DLgs 155/2010 Italy
1. Tzoutzas et al 56	Postgraduate clinic of the Dentistry School of the National and Kapodistrian University of Athens	PM _2,_ 5 = 2.05 to 72.63 g/m ^3^ with an average of 6.80 g/m ^3^ PM _10_ = 1.58 to 71.40 g/m ^3^ (mean 6.30 g/m ^3^ ). VOCs = 0.03 to 4.00 mg/m ^3^ with an average value of 0.79 mg/m ^3^ . CO2 = from 375 to 1,407 ppm (average 457 ppm). (all data were recorded continuously at 1-min intervals on a 24-h basis and were uploaded onto an online platform with the use of a wireless local area network)	PM _2,5_ = 15 mg/m ^3^ PM _10_ = 45 mg/m ^3^ VOCs = NA CO2 = 4 mg/m ^3^	PM _2,5_ = 25 mg/m ^3^ PM _10_ = 50 mg/m ^3^ VOCs = NA CO2 = NA	PM _2,5_ = – PM _10_ = 50 mg/m ^3^ VOCs = NA CO2 = NA
2. Liu et al 67	Dental Department of the Chang Gung Memorial Hospital in Taiwan	CO _2_ = ranged from 491.73 to 653.65 ppm TVOCs = the highest concentration occurred in GP (1,373.99 ppb) and the lowest occurred in the IR (674.56 ppb). PM = PM10 concentration was 75 μg − 3 for 24-h average concentration, while that for PM2.5 = was 35 μg/m for 24-h average concentration	CO2 = 4 mg/m ^3^ VOCs = NA PM _10_ = 45 mg/m ^3^ PM _2,5_ = 15 mg/m ^3^	CO2 = NA VOCs = NA PM _10_ = 50 mg/m ^3^ PM _2,5_ = 25 mg/m ^3^	CO2 = NA VOCs = NA PM _10_ = 50 mg/m ^3^ PM _2,5_ = –
3. Santarsiero et al 70	The Emergency Ward of the Ospedale Odontoiatrico George Eastman of Rome was chosen for the investigation	TVOCs = NA	VOCs = NA	VOCs = NA	VOCs = NA
4. Helmis et al 60	Dentistry Clinic, Dentistry Faculty, Athens University	COV total = 2,000–5,500 μg/m ^3^ (referred to maximum levels) CO _2_ = 1,500 ε4,600 mg/m ^3^ PM _10_ = 326 μg/m ^3^ PM _2,5_ = 205 μg/m ^3^ NO _2_ = 40–150 μg/m ^3^ SO _2_ = 5–30	VOCs = NA CO _2_ = 4 mg/m ^3^ PM _10_ = 45 mg/m ^3^ PM _2,5_ = 15 mg/m ^3^ NO _2_ = 10 mg/m ^3^ SO _2_ = 40 mg/m ^3^	VOCs = NA CO _2_ = NA PM _10_ = 50 mg/m ^3^ PM _2,5_ = 25 mg/m ^3^ NO _2_ = 40 mg/m ^3^ SO _2_ = 20 mg/m ^3^	VOCs = NA CO _2_ = NA PM _10_ = 50 mg/m ^3^ PM _2,5_ = – NO _2_ = 40 mg/m ^3^ SO _2_ =125 mg/m ^3^
5. Helmis et al 78	Two clinics of the Athens University Dentistry Faculty	TVOCs = 800 μg/m ^3^ CO _2_ = 2.100 mg/m ^3^ PM _10_ = 71 mg/m ^3^ PM2.5 = 36 mg/m ^3^ NO = 5–132 NO _2_ = 45–170 (μg/m ^3^ )	TVOCs = NA CO _2_ = 4 mg/m ^3^ PM _10_ = 45 mg/m ^3^ PM _2,5_ = 15 mg/m ^3^ NO = NA NO _2_ = 25	TVOCs = NA CO _2_ = NA PM _10_ = 50 mg/m ^3^ PM _2,5_ = 25 mg/m ^3^ NO = NA NO _2_ = –	TVOCs = NA CO _2_ = NA PM _10_ = 50 mg/m ^3^ PM _2,5_ = – NO = NA NO _2_ = –
6. Hong et al 62	Dental clinic in southern Taiwan	VOCs = 5.44 ppm (medium value) PM _10_ = 76 mg/m ^3^	VOCs = NA PM _10_ = 45 mg/m ^3^	VOCs = NA PM _10_ = 50 mg/m ^3^	VOCs = NA PM _10_ = 50 mg/m ^3^
7. Rexhepi et al 16	A dental unit located in an open plan clinic of the Department of Innovative Technologies in Medicine and Dentistry of the University “G. d'Annunzio” of Chieti-Pescara, Italy	PM1 = (open window) 34.49 ± 0.72 (close window) 20.73 ± 0.08 PM2,5 = (open window) 35.34 ± 0.76 (close window) 21.26 ± 0.08 PM10 = (open window) 44.66 ± 1.45 (close window) 25.01 ± 0.1	PM1 = NA PM _2,5_ = 15 mg/m ^3^ PM _10_ = 45 mg/m ^3^	PM1 = NA PM _2,5_ = 25 mg/m ^3^ PM _10_ = 50 mg/m ^3^	PM1 = NA PM _2,5_ = – PM _10_ = 50 mg/m ^3^

### Graphic Representation

**Fig. 3:**
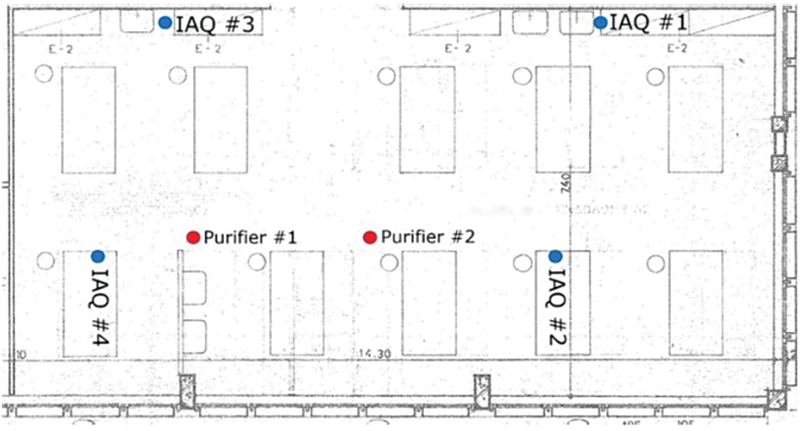
Positions of the IAQ sensors and the air purifiers in the clinic. 56

All the diagrams concerning the arrangement of purifiers and ventilation in dental clinics were reported, along with the six articles included in the systematic review (one had no graphic representations; Figs. 3 4 5 6 7 8 ). The only possible solution to date to reduce the rate of indoor pollution is to intensify mechanical and natural ventilation. 52 53 54

**Fig. 4:**
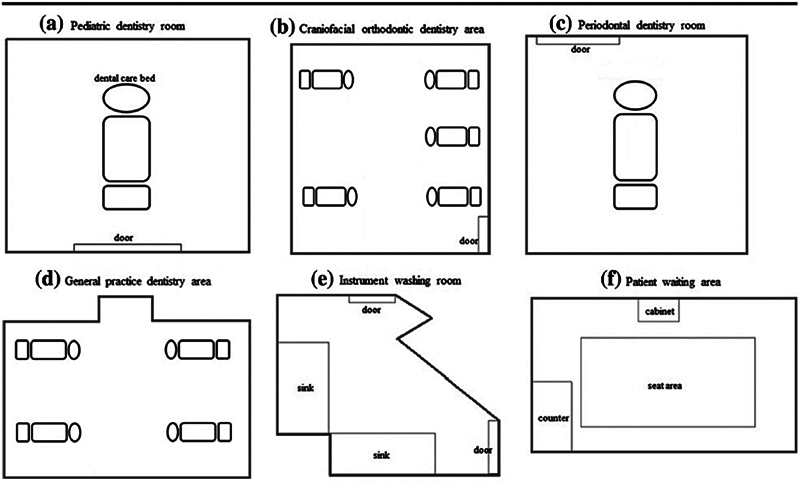
Diagram of the six locations of the dental department. 67

**Fig. 5:**
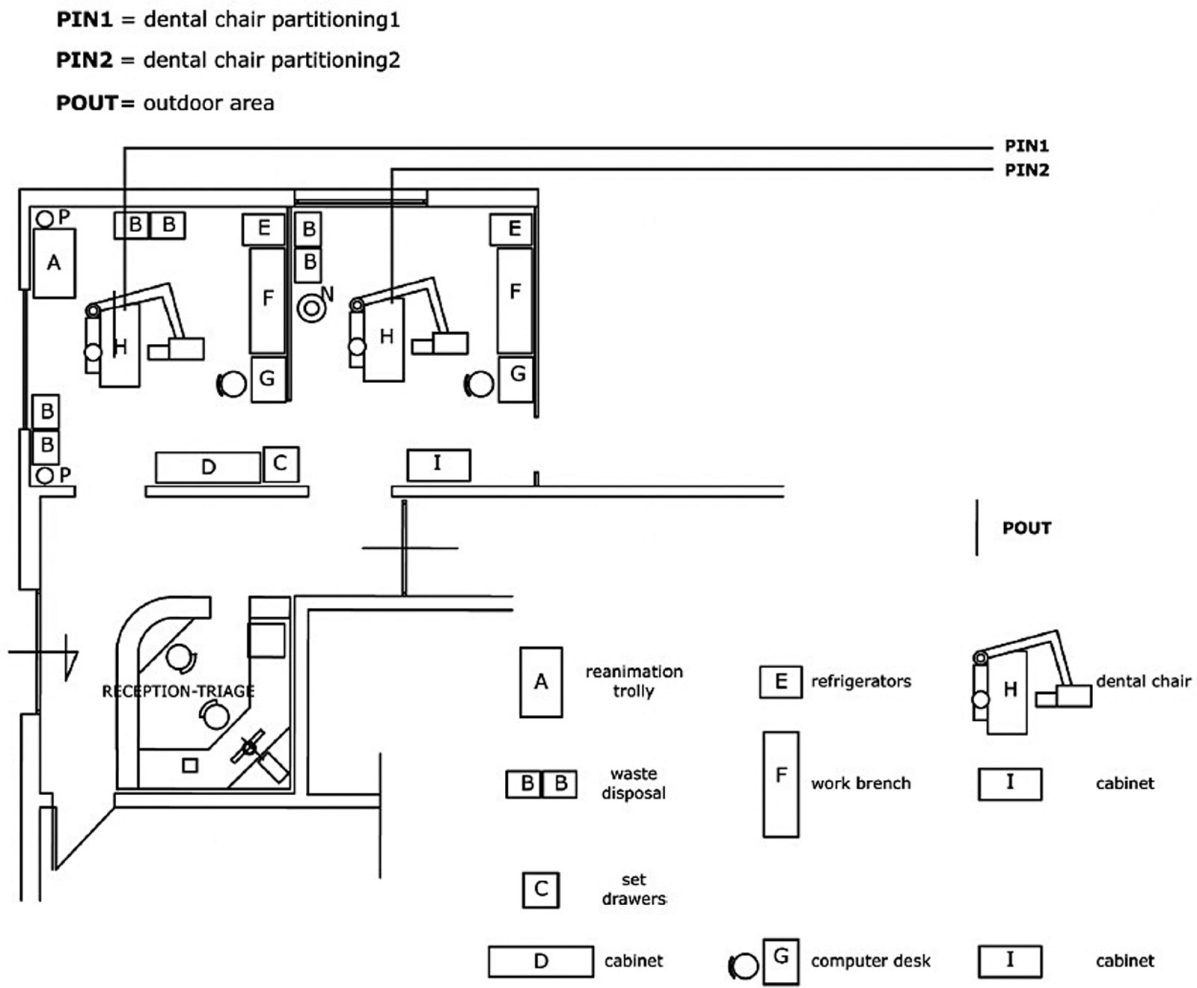
Reports the sampling point at the dental chair partitioning 1 (PIN1), the dental chair partitioning 2 (PIN2), and the outdoor window (POUT). 70

**Fig. 6:**
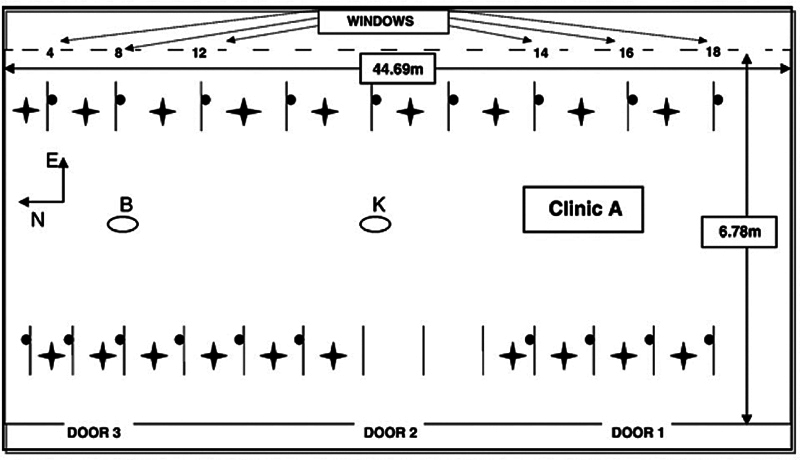
Ground plan of clinic, location of instruments for the pollutants measurements, and measurement points of the airflow characteristics (●: location of DANTEC instruments, *: dentistry chairs, |: bulkheads) (not in scale). 60

**Fig. 7:**
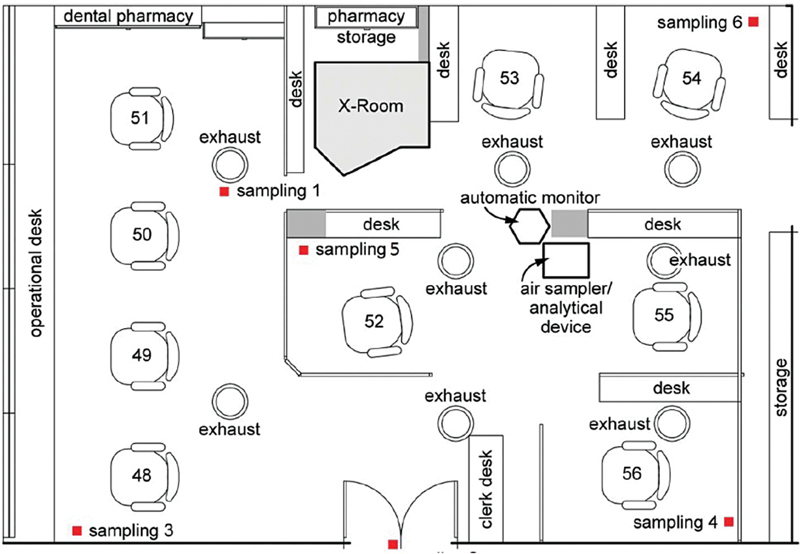
Layout of the dental clinic and air sampling locations, including sampling sites 1 and 2 for the control and sample sites 3, 4, 5, and 6 for the group studied. 78

**Fig. 8:**
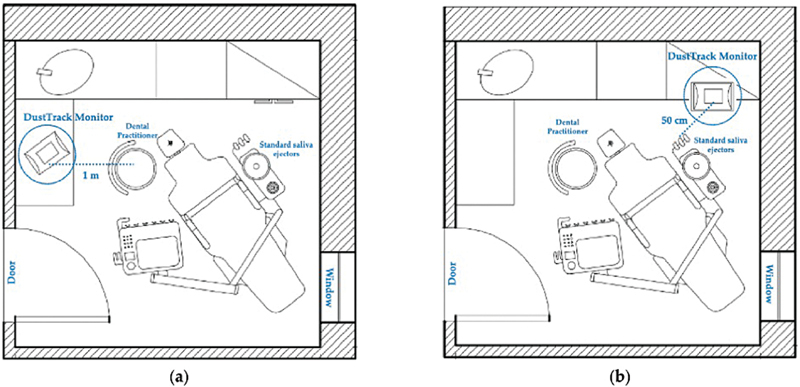
Position of the DustTrak DRX Aerosol Monitor in the plan of the dental unit in which the dental services were performed: ( **A** ) 1 m from the dental practitioner chair; ( **B** ) 50 cm from the standard saliva ejectors. 62

## Discussion

This systematic review aims to investigate the main pollutants present in dental practices and the reference levels existing in the literature, understand their characteristics, and identify their impact on the health of patients and healthcare workers. The ultimate goal is to develop strategies to reduce indoor pollution and improve air quality in dental practice.

Although the current guidelines on environmental pollutants are not entirely consistent and uniform, our analysis of the scientific literature has shown that temperature and humidity should be constant in the environments where measurements are taken and should fall within the limits set by the main guidelines, such as those of the American Society of Heating, Refrigerating and Air-Conditioning Engineers (ASHRAE). 55

In fact, Tzoutzas et al found that most observations (95%) fell within the measurement range known as the normal range of 23 to 26 °C. 56 All but two of the analyzed studies, which did not report temperature values, fall within this measurement range.

Furthermore, the authors emphasize the importance of air exchange through natural ventilation in environments with other infection risks, such as the dental sector, which affects temperature and atmospheric conditions, 16 and highlighted in all the studies analyzed, particularly in the two studies that examined the biological infection risk of COVID-19 as well. In addition, ventilation speed and direction associated with air pollution may be another factor that can affect the indoor environment. 57 58 59 However, the articles under review report that opening windows (i.e., natural ventilation) did not significantly affect the results obtained and that adequate mechanical ventilation must be used in synergy. 60 The scientific literature on the effects of ventilation on health, comfort, and productivity in non-industrial indoor environments (offices, schools, homes, etc.) has been reviewed by a multidisciplinary group of European scientists, called EUROVEN, with expertise in medicine, epidemiology, toxicology, and engineering. The research group agreed that ventilation is strongly associated with comfort (perceived air quality) and health (symptoms of sick building syndrome [SBS], inflammation, infections, asthma, allergies, etc.) and that there is an association between ventilation and productivity (office work performance). Finally, there may be an increased risk of SBS symptoms in air-conditioned buildings compared to naturally or mechanically ventilated buildings, and improper maintenance, design, and operation of air conditioning systems contribute to an increased prevalence of SBS symptoms. 8 19 20 21 22 23 24 25 26 27 28 29 30 31 32 33 34 35 36 37 38 39 40 41 42 43 44 45 46 47 48 49 50 51 52 53 54 55 57 58 59 61

Hong et al 62 found the highest average CO _2_ levels at 1,695.428 mg/m ^3^ , while others report CO2 levels in the range between 900 and 1,500 mg/m ^3^ , with the exception of Santarsiero et al who reported levels of other types of pollutants. This systematic review shows that CO _2_ levels are influenced by work activity (i.e., the presence or absence of people) and not by natural or mechanical ventilation. 63 64 65 This result was confirmed in the study by Fromme et al. 66 Furthermore, Liu et al highlighted that indoor pollutant levels are closely related to CO _2_ levels. In fact, when CO _2_ levels are reduced, TVOC and PM levels are lowered. 67 In 2023, the eighth report by the Lancet Countdown, an international research project that independently monitors the evolution of the health impacts of climate change and the emerging health benefits of climate action, brings together 114 scientists and health professionals from 52 research institutes and United Nations agencies around the world to provide the most comprehensive assessment to be improved. The report notes that 1,337 tons of CO _2_ are emitted globally every second and that every moment of delay in taking action increases the risks to human health and survival. 68

TVOC particles are strongly influenced by the type of activity conducted in dental practice, rather than by ventilation and occupancy. 69 In fact, they are associated with the biomaterials used in certain dental procedures. In support of this thesis, Liu et al found the highest levels of TVOC particles 674.56 ppb to 1,373.99 ppb of PM _(2.5)_ and TVOC 674.56 ppb to 1,373.99 ppb of PM _10_ in the prosthetics department, where resinous materials are used for the manufacture and relining of temporary restorations. Dimethyl methacrylate, due to its volatile properties, easily disperses into the air after the material has been polymerized and completely hardened. 67 The second most contaminated department was periodontology, as prosthetic work was being carried out on the days of the measurements. 67 Santarsiero et al detected high levels of TVOC released from materials such as formaldehyde, acetaldehyde, propionaldehyde, and isovaleric aldehyde. 70 The WHO has established an indoor air quality guideline for short- and long-term exposure to formaldehyde (FA) of 0.1 mg/m ^3^ (0.08 ppm) for all continuous exposure periods of 30 minutes. 71 This guideline has been supported by studies conducted from 2010 to 2013. Since 2013, new key studies have been published and important cancer cohorts have been updated, which we have evaluated and compared with the WHO guideline. FA is genotoxic, causes the formation of DNA adducts in human beings, and shows a clastogenic effect; nasopharyngeal cancer and leukemia have been observed inconsistently in different studies. New updates to the U.S. National Cancer Institute (NCI) cohort confirmed that the relative risk was not increased with average AF exposures below 1 ppm and peak exposures below 4 ppm. Hodgkin's lymphoma, which was not observed in the other studies reviewed, is not considered to be AF-dependent; it increased in the NCI cohort at average concentrations ≥0.6 mg/m ^3^ and peak exposures ≥2.5 mg/m ^3^ , both above the WHO guideline. Overall, the credibility of the WHO guideline has not been challenged by the new studies. 72 Helmis et al also suggested that the timing of powder/liquid mixing of resinous materials used in the prosthetics department should be extremely accurate. They point out that even detergents commonly used in dental practice disinfection release more TVOC particles; in fact, the highest levels of 75 μg/m ^3^ for PM _2.5_ and 138 μg/m ^3^ for PM _10_ were recorded during working hours, when prosthetic procedures were being performed, and at the end of the working day, when the main cleaning of the rooms is normally carried out. 60 The same authors recommend installing air purifiers in places where particles are emitted, as natural/mechanical ventilation is not sufficient to clean the environment. 73 The presence of pollutants has also been detected in ventilation filters. 74 Helmis et al confirm the same result, namely, that the prosthetics department, where procedures using resinous materials (in particular PMMA used in the production of posts and abutments) are performed, shows the highest levels of TVOC and exceeds the limits set by Greek legislation. Another solution suggested by the scientific literature is to improve the composition of cleaning products used in dentistry, using natural ingredients that are harmless to human health. 75

PM particles, on the other hand, are influenced by the external environment, ventilation, and the number of people circulating in the dental clinic.

Some studies have found associations between PM particles and fractional exhaled nitric oxide (FeNO), lung function, oxygen saturation, childhood asthma, and symptoms in patients with chronic obstructive pulmonary disease (COPD). High levels of VOCs have been associated with asthma and upper respiratory tract symptoms and, in severe cases, cancer. In the future, effective intervention studies for PM may focus on human behavior, along with air purifiers and increased ventilation, while interventions for VOCs may focus more on building materials and household products, along with cleaning and ventilation. 76

As shown in the results ( Fig. 1 ), PM particles have very harmful effects on human health, particularly for those who are exposed to them over the long term, such as healthcare workers in dental practice. 77

However, our article has several limitations: the articles analyzed are the most comprehensive on the subject, but they lack important information from a legislative point of view, such as whether they follow regional or national regulations, whether there is environmental monitoring by accredited institutions, or whether they follow international standards. There is a lack of information on the types of pollutants and the long-term effects on the health of operators after 20 to 30 years of occupation. Furthermore, the available data cannot be compared for a meta-analysis due to the above-mentioned limitations as well as geographical and climatic differences that significantly influence the results obtained. Finally, the ventilation used is not comparable, as most of the studies analyzed use different and combined types of natural and mechanical ventilation, as well as environmental filters, while some of them use natural ventilation exclusively.

Therefore, further clinical and laboratory studies are needed, such as cohort studies on respiratory outcomes or interventional trials with air-cleaning technologies, to test the levels of major indoor pollutants and the new technological tools recommended by scientific literature to address a well-established and serious human health concern affecting dental clinic healthcare workers.

## Conclusion

In consideration of the limits of this systematic review, some key points were identified that will be useful for further in vivo studies, aimed at developing specific guidelines to protect healthcare workers in dental facilities:

- Natural ventilation alone, or in combination with mechanical ventilation, is insufficient to ensure healthy indoor air quality in dental clinics. Therefore, ad hoc air purifiers and lamps must be used in areas where most of the polluting particles are released.
- Materials used in the prosthetic sector must be modified to incorporate eco-sustainable particles that are harmless to human health. The composition of detergents must be modified as well to prevent the release of particles polluting the air and settling on furniture surfaces and ventilation filters.
- Specific guidelines and international reference standards for indoor pollution in dental practice need to be developed to limit the harmful health effects caused by long-term exposure to indoor pollutants. The ultimate goal is therefore to be fully eco-sustainable for both the environment and the population, in line with the WHO's 2030 goals.
